# Multidrug Resistance and Plasmid Profiles of *Escherichia coli* Isolated from Lebanese Broiler Farms

**DOI:** 10.1155/2023/8811675

**Published:** 2023-06-01

**Authors:** Houssam Shaib, Paul Aoun, Ahmad Ghaddar, Hamza Al Labadi, Youssef Obeid

**Affiliations:** Department of Agriculture, Faculty of Agricultural and Food Sciences, American University of Beirut, Beirut, Lebanon

## Abstract

The present study was undertaken to determine the antimicrobial resistance patterns and plasmid fingerprints of commensal *Escherichia coli* isolated from Lebanese broiler chickens. To that end, a total of 30 *E. coli* isolates were collected from 15 semi-open broiler farms from North Lebanon and Bekaa Valley. Results showed that all the isolates were resistant to at least nine out of 18 evaluated antimicrobial agents. The best-performing antibiotic families were Carbapenems (Imipenem) and Quinolones (Ciprofloxacin and Norfloxacin) to which only 0.0 and 8.3% of the isolates were resistant, respectively. Fifteen various plasmid profiles were depicted, and all the isolates were found to possess one or multiple plasmids. The plasmid sizes varied from 1.2 to 21.0 kbp, and the most commonly detected plasmid had a size of 5.7 kbp (23.3% of the isolates). There was no significant association between the number of plasmids per isolate and resistance to a particular drug. Nevertheless, the presence of specific plasmids, namely, the 2.2 or 7.7 kbp sized ones, was strongly correlated to Quinolones or Trimethoprim resistance, respectively. Both the 7.7 and 6.8 kbp plasmids showed mild correlation to Amikacin resistance, and the 5.7 kbp plasmid was mildly correlated to Piperacillin-Tazobactam resistance. Our findings highlight the need to revise the list of antimicrobials currently used in Lebanese poultry and associate the presence of specific plasmids to antimicrobial resistance patterns in *E. coli* isolates. The revealed plasmid profiles could also serve any future epidemiological investigation of poultry disease outbreaks in the country.

## 1. Introduction

Growing rates of multidrug-resistant (MDR) bacteria pose a serious economic threat to the animal production sector and have major implications for public health. The increasing resistance to common antibiotics is mostly due to the chaotic utilization of antimicrobial agents to treat diseases and to promote the growth of farm animals [1, 2]. Exposure to antimicrobials of various groups can lead to cross-resistance, and the antibiotic resistance genes may spread among bacteria through horizontal gene transfer (HGT). That is, the antimicrobial resistance of commensal bacteria, such as *Escherichia coli*, is equally important as they constitute a reservoir and vector of resistance [3, 4]. When *E. coli* bacteria are in the presence of antibiotic pressure, they are forced to develop alternative ways to survive and grow in a harmful environment. That is why the resistance in *E. coli* has been increasing at a faster rate among livestock isolates than it did among human clinical isolates [5].

In fact, *E. coli* acquires, through HGT, genes that confer resistance to broad-spectrum Cephalosporins, Carbapenems, Aminoglycosides, Fluoroquinolones, and Macrolides among other antibiotic classes. Several studies have recently shown that the acquisition of resistance could be encoded by chromosomal or plasmid-mediated genes in bacteria. More specifically, plasmids are considered as the main vector in the dissemination of multidrug resistance through HGT [6, 7]. Plasmids carrying resistance genes can be interchangeably transferred among bacteria within the same or different species and genera by conjugation and natural transformation [7]. As a result, the treatment of *E. coli* infections has been complicated by the emergence and dissemination of plasmid-mediated resistance to Fluoroquinolones, Aminoglycosides, broad-spectrum Cephalosporin, Polymyxins, Sulfonamides, Tetracyclines, etc. [6, 8].

Several studies have identified multidrug-resistant *E. coli* isolated from poultry in Lebanon. They have also reported that *E. coli* originating from animal husbandry is a major biological contaminant of the Lebanese marine environment and water resources used for irrigation and drinking. In most of these studies, the major tools for genetic analysis were (1) the end point PCR, whereby selected genes were targeted, and (2) sequencing [9–19]. Although the said techniques are highly specific, plasmid fingerprinting (profiling) offers a relatively simple molecular typing method with low setup costs and provides a more practical tool for an insight on the role of plasmids in the transmission of antibiotic resistance in *E. coli,* among other bacteria [7, 20, 21]. However, the utility of plasmid profiling depends on multiple factors such as the variability of plasmid patterns within a species, the frequency of plasmid-free isolates, the stability of plasmids, and the reproducibility of plasmid patterns [22].

This study describes the antimicrobial resistance patterns of thirty commensal *E. coli* isolates isolated from selected Lebanese broiler farms and characterizes, for the first time, their plasmid fingerprints. It determines also the correlation between plasmid profiles and specific antimicrobial resistance patterns of the *E. coli* isolates. Moreover, the figures reported in this paper provide baseline data that would serve any epidemiological study to be carried out in the future.

## 2. Materials and Methods

### 2.1. Collection of *E. coli* Isolates

A total of 30 *E. coli* isolates were collected from 15 semi-open broiler farms distributed between two Lebanese districts, namely, North Lebanon and Bekaa. Intestinal samples from morbid or dead birds were acquired aseptically, cut open, and rinsed with sterile physiological saline (NaCl 0.9%). A loopful amount of the mucosal scraping of the intestinal sample was struck for isolation on MacConkey agar (Scharlau 02-418; Scharlau Chemie S.A., Barcelona, Spain) and incubated at 37°C for 24 h. Suspected *E. coli* isolates were further subcultured on another MacConkey plate. Lactose fermenting isolates were identified as *E. coli* following a biochemical identification process using the Analytical Profile Index 20E (API 20E) kit for *Enterobacteriaceae*. Confirmed *E. coli* isolates were preserved in Triple Sugar Iron Agar (TSI; Oxoid Ltd, Basingstoke, Hampshire, England) slants at −80°C until further analyses.

### 2.2. Antimicrobial Susceptibility Testing (AST)

*E. coli* isolates were inoculated into 5 mL of Tryptose Phosphate Broth (TPB; Oxoid Ltd, Basingstoke, Hampshire, England) and incubated at 37°C until Log phase was reached. The Log phase was identified whenever the turbidity of the bacterial suspension in TPB matched that of McFarland Standard #2 Barium Chloride suspension. An amount of 100 microliters of the bacterial suspension was then spread onto Mueller–Hinton Agar (MHA, Oxoid Ltd, Basingstoke, Hampshire, England) [23]. The antimicrobial disks (Oxoid Ltd, Basingstoke, Hampshire, England) used in the study were Ampicillin (AMP-10 *μ*g), Amoxicillin (AMC-20 *μ*g), Piperacillin-Tazobactam (TZP-100 *μ*g), Penicillin G (PG-10U), Cefoxitin (CTX-30 *μ*g), Cephalothin (KF-30 *μ*g), Fusidic Acid (FC-10 *μ*g), Aztreonam (ATM-30 *μ*g), Imipenem (IPM-10 *μ*g), Ciprofloxacin (CIP-5 *μ*g), Norfloxacin (NOR-10 *μ*g), Trimethoprim (TM-5 *μ*g), Sulfamethoxazole (STX-23.75 *μ*g), Tetracycline (T-30 *μ*g), Erythromycin (E-15 *μ*g), Amikacin (AK-30 *μ*g), Gentamicin (GM-30 *μ*g), and Clindamycin (CD-2 *μ*g). The plates were incubated overnight at 37°C, and the inhibition zone was subsequently observed and recorded. Interpretation charts were used to determine the susceptibility of *E. coli* isolate to each of the tested antimicrobials based on the inhibition zone diameter recorded.

### 2.3. Plasmid Profiling

Plasmid profiling of the *E. coli* isolates was performed as per the method described by Lonkar et al. [24]. Briefly, *E. coli* isolates were harvested at Log phase from 5 ml TPB culture by centrifugation at 2000 rpm for 10 min. The pellet was resuspended in 300 *μ*l of cold solution I (10 mmol·L^−1^ EDTA, 50 mmol·L^−1^ Tris, pH 7.5, 100 *μ*g·L^−1^ RNase A) and transferred in a 2 mL capacity microcentrifuge tube and vortexed for 1 min, at maximum speed. 300 *μ*l of solution II (0.2 mol·L^−1^ NaOH, 1% SDS) was added. After 5 min, 300 *μ*l of solution III (2.55 mol·L^−1^ potassium acetate, pH 4.8) was added. The tube was then centrifuged at 10,000 *g* for 15 min. Supernatant was transferred to a second microcentrifuge tube, and proteins were extracted with 600 *μ*l of a mixture of 25 : 24 : 1 (v/v) phenol-chloroform- isoamyl alcohol. After gentle shaking, the mixture was centrifuged at room temperature for 5 min at 10,000 *g*. The aqueous phase above the white protein interface was collected into a microcentrifuge tube, and the plasmid DNA was precipitated with one volume of isopropanol and pelleted by centrifugation at 4°C for 10 min at 15,000 g. The isopropanol was carefully removed, and the DNA pellets were dried. Plasmid DNA was dissolved in TE buffer composed of 20 *μ*l of 10 mmol·L^−1^ Tris, 1 mmol·L^−1^ EDTA pH 7.5. The DNA was examined by 1% agarose gel electrophoresis at 80 V for 2 hours. The kbp length of each plasmid was determined using the Quantity One software (BioRad).

### 2.4. Statistical Analysis

Pearson's correlation was used to test significant trends in the linear association of *E. coli* plasmid counts and antimicrobial resistance over a 95% confidence interval by using SPSS v. 25 system software (SPSS Inc). The same test was used to assess the strength and direction of the linear relationship between plasmids of specific kbp length and resistance to each of the evaluated antimicrobials. Strong positive correlation was determined based on *P* value and Pearson's coefficient (*R*) whereby *P* should be less than 0.001 and *R* should be greater than 0.7.

## 3. Results

### 3.1. Antimicrobial Susceptibility Testing (AST)

**Figure 1:**
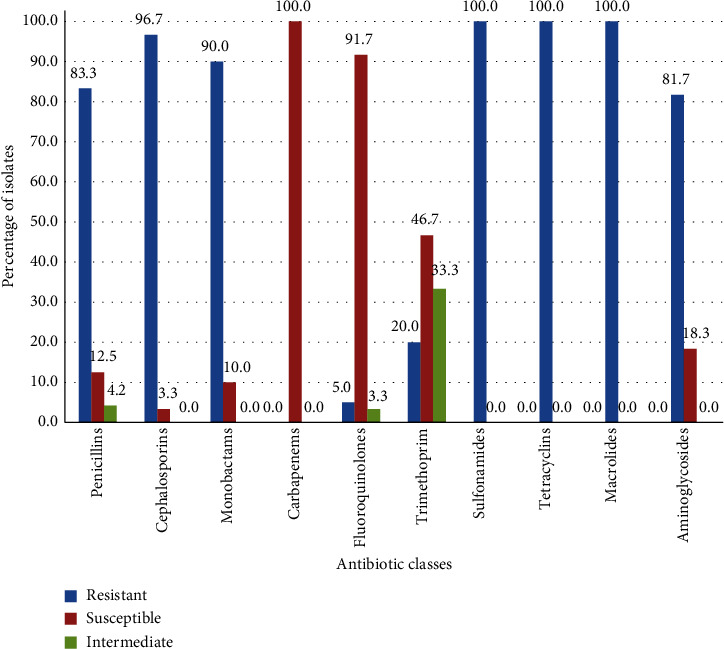
Resistance percentage of thirty commensal *E. coli* isolates isolated from broiler intestinal specimens against various classes of antimicrobials.

In this study, a total of thirty different *E. coli* isolates were retrieved from intestinal samples collected from 15 broiler farms. The resistance of these isolates was tested against 18 antibiotics belonging to 10 different major antimicrobial classes. Each isolate was resistant to at least 11 different antimicrobial agents out of 18. As shown in Figure 1, the highest resistance rate was recorded against three antimicrobial classes, namely, Sulfonamides, Tetracyclines, and Macrolides (100.0%) followed by Cephalosporins (96.7%), Monobactams (90.0%), Penicillins (83.3%), and Aminoglycosides (81.7%). The least resistance rate was observed against Fluoroquinolones (5.0%) and Carbapenems (0.0%). When taken individually, the best active antibiotics were Imipenem, Norfloxacin, and Ciprofloxacin to which the *E. coli* isolates exhibited 0.0, 3.3, and 6.7% resistance, respectively, while the least active were Penicillin G, Cephalothin, Fusidic Acid, Sulfamethoxazole, Tetracycline Erythromycin, Gentamicin, and Clindamycin with 100.0% *E. coli* resistance (Table 1).

### 3.2. Plasmid Profiling and Its Correlation with Antimicrobial Resistance

**Figure 2:**
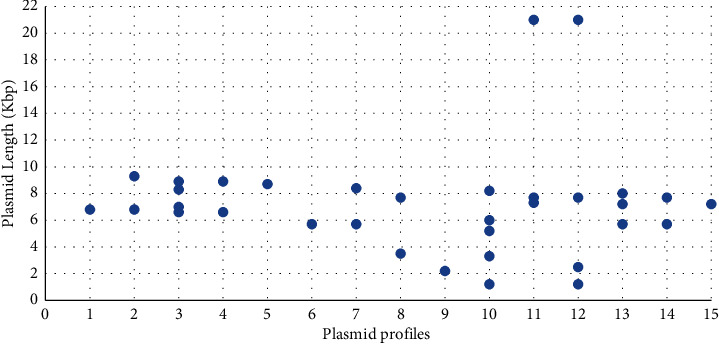
Different plasmid profiles of commensal *E. coli* isolates obtained in this study.

At least one plasmid was detected in all the *E. coli* isolated in this study. Thirty six percent of the isolates had one plasmid while 33.3% harbored two. The rest exhibited three (10%), four (13.3%), or five (3.3%) plasmids. The depicted plasmids showed a length that was varying between 1.2 and 21.0 kbp (Table 2), and the most commonly detected plasmid (23.3% of the isolates) had a size of 5.7 kbp. Overall, 15 different plasmid profiles were detected as shown in Table 2 and Figure 2 and were labelled 1 through 15.

Remarkably, even the isolates that had the same plasmid profile did not always demonstrate the same antimicrobial resistance pattern as it is the case of isolate 1 vs. isolates 2, 3, and 4. The same applies for isolates 15 through 19 that had the same plasmid fingerprinting profile, yet only isolates 18 and 19 had similar antimicrobial resistance pattern in comparison to the others (isolates 15, 16, and 17). Moreover, and despite the diversity of the plasmid profiles recorded, all the *E. coli* isolates were resistant to 9 different antibiotics, namely, Ampicillin (AMP), Cephalothin (KF), Sulfamethoxazole (SMX), Tetracycline (T), Erythromycin (E), Fusidic Acid (FC), Gentamicin (GM), Penicillin G (PG), and Clindamycin (CD).

The antimicrobial resistance profile was not correlated to the number of plasmids detected in the commensal *E. coli* isolates obtained in this study (*R* = −0.231, *P* > 0.05). In addition, most of the detected plasmids were not significantly correlated to resistance to any specific antibiotic among the evaluated ones. Most importantly, few plasmids exhibited strong or mild correlation to specific antimicrobial resistance (Table 3). For instance, resistance to Fluoroquinolones (CIP and NOR) was strongly correlated to the presence of the 2.2 kbp plasmid (*R* = 1, *P* ≤ 0.001) while resistance to TM was strongly correlated to the presence of the 7.7 kbp plasmid (*R* = 0.882, *P* ≤ 0.001). On the other hand, resistance to AK was mildly associated to the presence of either 6.8 or 7.7 kbp plasmids (*R* = 0.363, *P* ≤ 0.05). One plasmid was also mildly correlated to TZP resistance, namely, the 5.7 kbp with an *R* value of 0.471 (*P* ≤ 0.05).

## 4. Discussion

The rapid surge in the development of bacterial resistance to antibiotics is a major concern and an issue of public health interest. In fact, the excessive use of antimicrobial agents in animal husbandry is one of the major contributors to the emergence of resistant strains worldwide whereby antibiotics are used for therapeutic treatment and as growth promoter feed additives. The alarming results obtained in this study made no exception. In fact, all the commensal *E. coli* isolated from broilers in this work are multidrug-resistant (MDR) bacteria which means that they are resistant to antibiotics that belong to at least three different antimicrobial classes [25]. This reflects the deleterious impact of continuous overuse of antibiotics in the poultry sector in Lebanon including Ampicillin, Gentamicin, Tetracycline, Erythromycin, and Doxycycline. Our findings agree with abundant studies on multidrug-resistant bacteria isolated from Lebanese poultry. For instance, many authors [10, 12–14, 16] reported resistance of commensal *E. coli* isolated from broilers to Penicillin, Ampicillin, Cefepime, Cefotaxime, Levofloxacin, Doripenem, Cefixime, Gentamicin, Kanamycin, Streptomycin, Tetracycline, and Sulfamethoxazole/Trimethoprim among other antibiotics. Remarkably, the same authors also documented a frequent bacterial resistance to Fluoroquinolones (CIP and NOR) which oppose the findings of this study. The fluctuating results could be linked to the complexity of resistance acquisition process against Fluoroquinolones. It is well known that the development of bacterial resistance to Quinolones is a multifactorial process whereby genes of both chromosomal and plasmid origin are involved [26]. As a matter of fact, the resistance of *Enterobacteriaceae* to Quinolones needs a sequence of several mutations: (1) a single mutation in the gyrA gene confers low‐level Quinolone resistance; (2) the acquisition of a second mutation either in the amino acid codon Ser‐80 or in the amino acid codon Glu‐84 of the parC gene confers a moderate level of Ciprofloxacin resistance; (3) a third mutation, the second in gyrA, leads to a high level of Ciprofloxacin resistance; and (4) a fourth mutation, the second in parC, confers the highest level of resistance [27]. It is worth noting that Quinolones, namely, Ciprofloxacin, had the highest occurrence percentage (32.5%) in chicken carcasses collected from the Lebanese market among four antibiotics families, as revealed in the work of Jammoul and El Darra [28]. This is one of the indications that Quinolones are among the most frequently used antibiotics in Lebanese poultry husbandry.

All the isolates were susceptible to Imipenem. Similar results were obtained by Mikhayel et al. [14] where bacteria of the *Enterobacteriaceae* family isolated from rectal swabs of poultry did not exhibit resistance to Imipenem. Kassem et al. [10] reported inconsistent susceptibility of *E. coli* isolated from poultry to Carbapenems, and the isolates that were resistant to Doripenem carried a plasmid-mediated gene (blaCMY-2) that confers resistance to Carbapenems in porin-deficient strains. The absence of resistance to Imipenem in this study and in the works of Mikhayel et al. [14] might be reassuring for the time being as it is considered, along with Colistin, one of the last resort antibiotics for the treatment of human infection with MDR Gram-negative bacteria [29]. However, *E. coli* resistance to Doripenem, reported in the work of Kassem et al. [10], might raise a red flag vis-a-vis the use of Carbapenems in controlling Gram-negative bacterial infections in the future.

In regard to plasmid profiles, the number of plasmids/isolate varied between one and five, with kbp length varying between 1.2 and 21.0. A total of 15 plasmid profile patterns were recorded for the 30 commensal *E. coli* isolates. The perfect situation would be to show that all of the isolates that have a specific antibiogram profile would have the same plasmid number and fingerprint. However, the number of plasmids/isolate was found to have no significant correlation to antimicrobial resistance. There is abundant literature that reports the same findings whereby resistance to antibiotics is related more to the presence of specific plasmids rather than their number [30–33]. The reason could be that plasmids have numerous functions besides antimicrobial resistance such as fertility plasmids (F), virulence plasmids, degradative plasmids, and Col plasmids responsible for the production of bacteriocin (colicin). That is, a higher number of plasmids would not entail automatically higher resistance to antibiotics, especially in Gram-negative bacteria where most of the said plasmids have been reported [34]. The presence of multiple resistance on a single plasmid or multiple plasmids in the same organism might also explain the lack of significant correlation between the plasmid number and the *E. coli* resistance pattern [35]. In addition, the role of integrons in bacterial exchange of genes between its chromosome and plasmids is vital in the acquisition and dissemination of resistance genes. Consequently, the dynamics of these integrons might further explain the absence of a significant association between the multidrug resistance of *E. coli* isolated in this study and the number of plasmids per isolate [36]. Remarkably, all of the isolates carried at least one plasmid which prohibited the comparison of the resistance pattern between plasmid-bearing and plasmid-free* E. coli* isolates.

This study reveals a solid relationship between the presence of particular plasmids and resistance to antimicrobial agent(s). The strong correlation between the presence of the 2.2 kbp plasmid and the resistance to Quinolones evaluated in this study (CIP and NOR) indicates that even small plasmids could be carriers of specific resistance patterns [37–39]. Although these small plasmids are most likely not self-transmissible due to the lack of conjugative genes, the presence of other large plasmids could fill the gap and help transfer small plasmids and other short transmissible elements (STEs) horizontally from one bacterium to another [40]. This study also reported a strong correlation between the presence of a 7.7 kbp plasmid in *E. coli* and resistance to Trimethoprim (TM). Several studies have linked TM resistance in *E. coli* isolates to large plasmids that could have a size of up to 180 kbp. A single large plasmid can carry resistance genes to many other antibiotics at the same time such as Chloramphenicol, Tetracyclines, Aminoglycosides, Quinolones, and Sulfonamides [8, 37]. Apparently, smaller plasmids would carry resistance traits against very few antibiotics as in the case of the 7.7 kbp revealed in this study and which is also mildly correlated to AK resistance along with the 6.8 kbp one. Actually, there is abundant literature correlating AK resistance in *E. coli* to the presence of specific genes in small plasmids (less than 13 kbp) that carry genes encoding for Amikacin phosphotransferases and adenylyltransferases. Moreover, and despite the fact that small plasmids carry very few genes, their presence play a significant role in plasmid evolution through processes mediated by mobile elements and mechanisms of recombination with other plasmids leading to an increased resistance to several antibiotics including Amikacin [41–43]. Piperacillin/Tazobactam (TZP) resistance in the studied *E. coli* isolates was mildly correlated to another small plasmid having a length of 5.7 kbp. In general, the predominant cause of resistance to *β*-lactam antibiotics, including TZP, in Gram-negative bacteria is mostly related to the hyperproduction of plasmid-mediated TEM-1 *β*-lactamases, production of extended-spectrum beta-lactamases (ESBLs), production of AmpC enzymes which are encoded by plasmid genes, and Carbapenem-hydrolyzing *β*-lactamases [44–46]. The plasmid-mediated TZP resistance does not necessarily dictate the presence of large plasmids, as revealed in this study. These results are in agreement with those of Hubbard et al. [47] who reported that a small circular DNA of around 10.9 kbp was found in TZP-resistant *E. coli* isolates. The said authors revealed that the large plasmids that were present in the TZP-resistant strains did not contain any antimicrobial or heavy metal resistance genes. However, the characterization of the small circular DNA molecule showed that it contained the missing TZP resistance genes along with several putative transposable elements.

The correlation results reported in this study indicate a strong association between specific plasmids in commensal *E. coli* isolated from poultry and resistance to particular antibiotics. However, this can be also very useful when conducting epidemiological surveillance and consequently developing strategies to curb the spread of plasmid-borne bacterial resistance. The findings could have been confirmed through conjugation studies whereby a better understanding of resistance would have been achieved by predicting the transfer of antimicrobial resistance-mediated conjugative plasmids. Moreover, plasmid sequencing could have unveiled key plasmid-specific functions such as conjugative ability, replication, and mobility. This would have enabled the classification of the plasmids reported in this study into various categories based on their phylogenetic relatedness and provided insight into the epidemiology of plasmid-mediated antimicrobial resistance of commensal *E. coli* isolated from poultry.

## Figures and Tables

**Table 1:** Resistance pattern of *E. coli* isolated from poultry intestinal specimens to various antimicrobials evaluated in this study.

Antibiotic	Antibiotic class	% of isolates		
		Resistant	Intermediate	Susceptible
AMP (Ampicillin)	Penicillins	100.0	0.0	0.0
AMC (Amoxicillin)	Penicillins	93.3	6.7	0.0
TZP (Piperacillin + Tazobactam)	Penicillins	40.0	10.0	50.0
PG (Penicillin G)	Penicillins	100.0	0.0	0.0
CTX (Cefoxitin)	Cephalosporin 2	90.0	0.0	10.0
KF (Cephalothin)	Cephalosporin 1	100.0	0.0	0.0
FC (Fusidic Acid)	Cephalosporin P1	100.0	0.0	0.0
ATM (Aztreonam)	Monobactam	90.0	0.0	10.0
IPM (Imipenem)	Carbapenem	0.0	0.0	100.0
NOR (Norfloxacin)	Fluoroquinolones	3.3	6.7	90.0
CIP (Ciprofloxacin)	Fluoroquinolones	6.7	0.0	93.3
TM (Trimethoprim)	Trimethoprim	20.0	33.3	46.7
SMX (Sulfamethoxazole)	Sulfonamides	100.0	0.0	0.0
T (Tetracycline)	Tetracyclines	100.0	0.0	0.0
E (Erythromycin)	Macrolides	100.0	0.0	0.0
AK (Amikacin)	Aminoglycoside	63.3	0.0	36.7
GM (Gentamicin)	Aminoglycoside	100.0	0.0	0.0
CD (Clindamycin)	Macrolides	100.0	0.0	0.0

**Table 2:** Plasmid profiles and resistance pattern of commensal *E. coli* isolates.

Profile	Number of plasmids	Plasmid size (kbp)	Frequency of bacteria exhibiting pattern (%)	Resistance pattern^*∗*^
1	1	6.8	4 (13.3)	Isolate 1: CTX, ATM, AK, AMC, TM Isolates 2, 3, and 4: CTX, ATM, AK, AMC
2	2	6.8, 9.3	1 (3.3)	Isolate 5: CTX, ATM, AK, AMC
3	4	6.6, 7.0, 8.3, 8.9	3 (10.0)	Isolates 6, 7, and 8: AK, AMC
4	2	6.6, 8.9	1 (3.3)	Isolate 9: CTX, ATM, AK, AMC
5	1	8.7	4 (13.3)	Isolates 10, 11, 12, and 13: CTX, ATM, AMC
6	1	5.7	1 (3.3)	Isolate 14: CTX, TZP, AK, AMC
7	2	5.7, 8.4	5 (16.7)	Isolate 15: CTX, AK, AMC Isolate 16: CTX, ATM, TZP, AK, AMC I solate 17: ATM, TZP, AMC Isolates 18 and 19: CTX, ATM, TZP, AMC
8	2	3.5, 7.7	2 (6.7)	Isolates 20 and 21: CTX, ATM, AMC
9	1	2.2	1 (3.3)	Isolate 22: CTX, CIP, ATM, NOR, TZP, AK, AMC, TM
10	5	1.2, 3.3, 5.2, 6.0, 8.2	2 (6.7)	Isolates 23 and 24: CTX, ATM, TZP, AMC
11	3	7.3, 7.7, 21.0	1 (3.3)	Isolate 25: CTX, ATM, TZP, AK, AMC
12	4	1.2, 2.5, 7.7, 21.0	1 (3.3)	Isolate 26: CTX, ATM, TZP, AK, TM
13	3	5.7, 7.2, 8.0	2 (6.7)	Isolates 27 and 28: CTX, ATM, TZP, AK,
14	2	2.2, 7.7	1 (3.3)	Isolate 29: CTX, CIP, ATM, AK, AMC, TM
15	1	7.7	1 (3.3)	Isolate 30: CTX, ATM, AMC, TM

^*∗*^All the isolates are also resistant to Ampicillin (AMP), Cephalothin (KF), Sulfamethoxazole (SMX), Tetracycline (T), Erythromycin (E), Fusidic Acid (FC), Gentamicin (GM), Penicillin G (PG), and Clindamycin (CD).

**Table 3:** Correlation between the presence of specific *E. coli* plasmids and antimicrobial resistance.

Plasmid length (kbp)	Potential resistance to	Pearson correlation coefficient	*P* value	Correlation type
2.2	CIP, NOR	1.000	≤0.001	Strong
7.7	TM	0.882	≤0.001	Strong
7.7	AK	0.363	0.045	Mild
5.7	TZP	0.471	0.036	Mild
6.8	AK	0.363	0.045	Mild

## Data Availability

The datasets used and/or analyzed during this study are available from the corresponding author on reasonable request.
